# Edge betweenness centrality as a failure predictor in network models of structurally disordered materials

**DOI:** 10.1038/s41598-022-15842-y

**Published:** 2022-07-12

**Authors:** Mahshid Pournajar, Michael Zaiser, Paolo Moretti

**Affiliations:** Department of Materials Science, WW8-Materials Simulation, Friedrich-Alexander Universität Erlangen-Nürnberg, Fürth, 90762 Germany

**Keywords:** Complex networks, Materials science, Statistical physics, thermodynamics and nonlinear dynamics

## Abstract

Network theoretical measures such as geodesic edge betweenness centrality (GEBC) have been proposed as failure predictors in network models of load-driven materials failure. Edge betweenness centrality ranks which links are significant, based on the fraction of shortest paths that pass through the links between network nodes. We study GEBC as a failure predictor for two-dimensional fuse network models of load transmission in structurally disordered materials. We analyze the evolution of edge betweenness centrality in the run-up to failure and the correlation between GEBC and failure propensity for both hierarchical and non-hierarchical networks exhibiting various degrees of disorder. We observe a non trivial relationship between GEBC and failure propensity, which suggests that the idea of GEBC as a useful failure predictor needs to be strongly qualified.

## Introduction

The identification of failure locations in materials is a generic problem in engineering mechanics. In a perfect material, failure is controlled by the largest stress concentration. Real materials are not perfect. They are disordered, and therefore in fracture of a real material fluctuations and statistical considerations play a major role. Thus, the question which we have to address in a disordered material is the following: In which sense are the locations of catastrophic failure initiation, or of local damage accumulation, different from the rest of the material?

In this research work, we explore a method to predict failure locations in quasi-brittle materials by using topological measures to distinguish locations of enhanced local failure probability. In ideal elastic-brittle materials, failure occurs by nucleation and growth of a crack that separates the sample along a failure surface, thus, failure occurs strictly at the location of the largest stress concentration at the crack tip. In quasi-brittle materials, by contrast, damage accumulation is spread over a fracture process zone ahead of the crack tip. This behavior emerges as a consequence of disorder, which in this case refers to local fluctuations in failure thresholds. In materials with large disorder, this process zone may be extensive and increasing the size of process zone leads to a transition from localized to diffuse failure. Thus, statistical measures are required for failure prediction^1–3^.

Many quasi-brittle materials are also microstructurally disordered, i.e., characterized by heterogeneity in their structural arrangement. Recently, disordered mechanical meta-materials of this type have been developed, which exhibit remarkable properties such as high strength to weight ratio and auxetic behavior^4^. Mechanical properties and failure behavior of materials can, in fact, be controlled by tuning only topology and geometrical structure rather than material properties^5^. Hanifpour et al. showed that the mechanical properties and fracture mechanisms of disordered lattices are dependent on geometry^6^. They have shown that the topology of the lattice is crucial for the auxetic behavior of materials with negative Poisson’s ratio as even small changes in topology can significantly affect the Poisson’s ratio. More generally speaking, controlling geometry and topology is at the core of designing metamaterials with tailored mechanical response^7^.

In relation to tuneable failure properties, it has been shown that material strength and toughness can be improved by endowing materials with appropriately designed hierarchical (micro)structures^8,9^. Sen and Buehler argue that through a hierarchical structure, mechanical properties of brittle materials can be improved to enhance fracture toughness^10^. Fuse- and beam network models have been used to analyze the effect of hierarchical organization on damage accumulation and modes of failure^11–13^. Results indicate that hierarchical (micro)structure affects failure considerably, by suppressing crack propagation in favor of local damage nucleation and diffuse percolation.

The effect of structural and geometrical properties on failure mechanisms can be investigated via network analysis approaches. Network analysis can be applied for analyzing various types of materials and structures that are representable as networks carrying loads^14–16^. The applicability of this method is not merely limited to systems that are topologically structured as networks of edges but may also encompass analysis of bulk material properties, including porous materials^17^ and biological matter^18^. There are various studies which apply network analysis methods to study technological infrastructures such as the internet, electrical supply networks or transportation networks. In view of the stability of such networks, nodes with large betweenness centrality seem to be key features of the investigated systems^19,20^. Edge Betweenness Centrality (EBC) is a measure describing the frequency at which an edge lies on the shortest path between pairs of nodes in a network (for a mathematical definition, see our methods section). In the context of materials design, it has been proposed that potential failure locations can be identified by correlation to large values of edge betweenness centrality^21^. Recent studies seem to confirm that material failure occurs preferentially at locations, which exhibit large Geodesic (i.e., strictly relying on the shortest path metric) Edge Betweenness Centrality (GEBC) values^22,23^. These results demonstrate that assessing failure locations of a system not necessarily requires the calculation of the local loadings, e.g. in terms of locally stored elastic energy. Analogously, the relevance of the shortest-path metric and of the GEBC has been pointed out in problems of force transmission^24^, heat conduction^25^, and transport phenomena^26^.

Predicting failure locations, however, is inherently more complex than establishing that links that fail have high centrality, as mechanical failure is contingent on the interplay of local and global stress patterns and their evolution under load, and of local material properties and damage evolution^27–31^.

The goal of our study is establishing the usability of the GEBC metric as a structural predictor of future failure events, in models of quasi-brittle brittle materials of varying degrees of local-strength disorder. In particular, we consider both hierarchical and non-hierarchical structures. What network metrics set apart future failure locations before damage takes place? Do predictions improve as damage is accumulated and failure approaches? To answer these questions, we simulate loading and failure in our model, using the Random Fuse Model (RFM)^32–34^. We calculate GEBC of elements in the initial state of both hierarchical and random networks, investigate how GEBC statistics changes until global failure, and how these changes correlate with the propensity of single network links to fail at a certain stage of the damage accumulation process.

## Methods

In the following, we introduce the methods that we have adopted in our analysis. The table below contains the definitions of the main symbols and acronyms that we use in the rest of the paper, grouped thematically.

**Table:** 

GEBC	Geodesic Edge Betweenness Centrality
RFM	Random Fuse Model
HFN	Hierarchical Fuse Network
SHFN	Shuffled Hierarchical Fuse Network
RFN	Random Fuse Networks
*C*	GEBC of a given edge
*N*	Number of nodes in a network
*E*	Number of edges in a network
*L*	Linear size of a system
*n*	Number of hierarchical levels
(*ij*)	Generic edge connecting nodes *i* and *j*
$V_i$	Voltage (displacement) at node *i*
$I_{ij}$	Current (force) at edge (*ij*)
$t_{ij}$	Current threshold of edge (*ij*)
$\eta$	Failing edge index
$\beta$	Failing edge index, counting from failure
$N_\mathrm {f}$	Final number of failed edges
$(ij)_\eta$	$\eta$-th failing edge
$I_\eta$	Global current at failure stage $\eta$
$V_\eta$	Global voltage at failure stage $\eta$
$f_\eta$	Maximum current-strength ratio at failure stage $\eta$
$\varepsilon$	Global strain
$\sigma$	Global stress
$\sigma _\mathrm {p}$	Peak stress
$\varepsilon _\mathrm {f}$	Failure strain
$\varepsilon _\mathrm {p}$	Strain at peak stress
$\alpha$	Failure class
*z*	Number of edges per sample in a failure class
$\Sigma _C^\alpha$	GEBC mean deviation for class $\alpha$

### Geodesic edge betweenness centrality

GEBC is a network theoretical measure to characterize the relevance of edges for the transport properties of a network, and also to identify community boundaries in network structures. We consider a network consisting of *N* nodes connected by *E* edges. Our network is undirected (each edge can be traversed in both directions) and unweighted (all edges count as a step of unit length along a path). Each edge connecting generic nodes *i* and *j* is identified by a generic index *h*, and its end nodes by the the ordered pair (*ij*). Under these assumptions, we compute path length simply as the number of edges along the path. The GEBC value *C*(*h*) of an edge *h* is then defined as

1 $$\begin{aligned} C(h) = \frac{2}{N(N-1)}\sum _{a\ne b} \frac{\sigma _{ab}(h)}{\sigma _{ab}} \end{aligned}$$

where , $\sigma _{ab}$ is the number of all shortest network paths connecting nodes *a* and *b*, and $\sigma _{ab}(h)$ is the number of all of these paths that pass through edge *h*.

In networks with a community structure, edges that connect different communities have high GEBC values since all of the shortest paths connecting nodes from the respective communities pass through those links. Therefore, by removing such edges, different communities of the network are separated from each other^35^.

From a computational perspective, direct implementation of Eq. (1) is prohibitively expensive for large networks, hence we use the algorithm formulated by Brandes^36^ in actual computations.

### Construction of hierarchical (HFN) and non-hierarchical (RFN) networks

Fuse network models provide a computationally efficient way of studying load-driven failure processes. Such models consider networks of nodes connected by load-carrying edges. Each node *i* is associated with a scalar displacement-like variable (“voltage”, or “strain”) $V_i$ while the connecting edges, which are assumed of unit length, are associated with scalar load variables (“currents”, or “stresses”): an edge (*ij*) connecting nodes *i* and *j* is envisaged as an ohmic resistor of unit conductance which, under a voltage difference between the two nodes, carries a current $I_{ij} = V_i - V_j$. Coupled Kirchhoff equations for all nodes are solved to compute the global current/voltage pattern. Once the current through an edge reaches a certain threshold, $|I_{ij}|\le t_{ij}$, the conductance of the edge is set to zero – in the electrical analogue, the fuse burns. Such fuse network models are characterized by the topology of the network on the one hand, and by the physical properties of the edges (conductances and failure thresholds) on the other hand.

In the present study, we shall assume that all edges have statistically equivalent properties, with unit length and unit conductance. Threshold currents are independent identically distributed random variables, which are assigned to the edges according to a Weibull distribution with unit mean^37,38^ i.e., the cumulative distribution function is

2 $$\begin{aligned} P(t_{ij}) = 1- \exp \left[ -\left( \frac{t_{ij}}{t_0}\right) ^k\right] , \quad t_0=1/\Gamma (1+1/k). \end{aligned}$$

The Weibull shape parameter *k* (“Weibull exponent”) controls the statistical spread of the values of $t_{ij}$, with larger values of *k* pointing to narrower distribution. The choice of the Weibull distribution is common in the materials science literature and is motivated by physical reasoning^34,39^. Each edge can be considered a one-dimensional assembly of load carrying elements of heterogeneously distributed strengths. The global strength of the edge can thus be computed using arguments of statistics of extremes, and under relatively general assumptions $t_{ij}$ follows a Weibull distribution such as in Eq. (2). As customary in statistical models of materials failure, the statistical spread of threshold values $t_{ij}$ is a way to model local heterogeneity of a material, and thus “disorder”. The use of Weibull distributions allows us to tune disorder, by acting on *k*: systems with $k\approx 1$ are highly disordered, whereas larger values of *k* point to a more homogeneous local strength distribution, and thus less disorder. We note that other definitions of disorder are possible. For instance, disorder may refer to topological heterogeneity (rather then response heterogeneity), as encountered in models for rod- and nanowire networks^40–42^.

In terms of geometry, we consider two-dimensional (2D) networks where we distinguish a loading direction (in the following, “vertical” direction) and a perpendicular direction (in the following, “horizontal” direction). The models are based on a square lattice of nodes sandwiched between top and bottom bus bars which ensure a constant potential difference across the network in the loading direction. The bus bars are connected by $L=2^n$ vertical columns consisting of $L-1$ nodes connected by *L* edges. Vertical edges are in the following denoted as load carrying edges, while the columns are denoted as load carrying fibers. In perpendicular direction, the load carrying fibers are connected by horizontal edges between adjacent nodes (denoted as “cross links”) to form a network structure. We consider two types of cross link patterns which lead to different global network topologies, henceforth denoted as hierarchical (HFN) and random (RFN) networks. We first deal with the HFN case.

#### Hierarchical network construction

**Figure 1:**
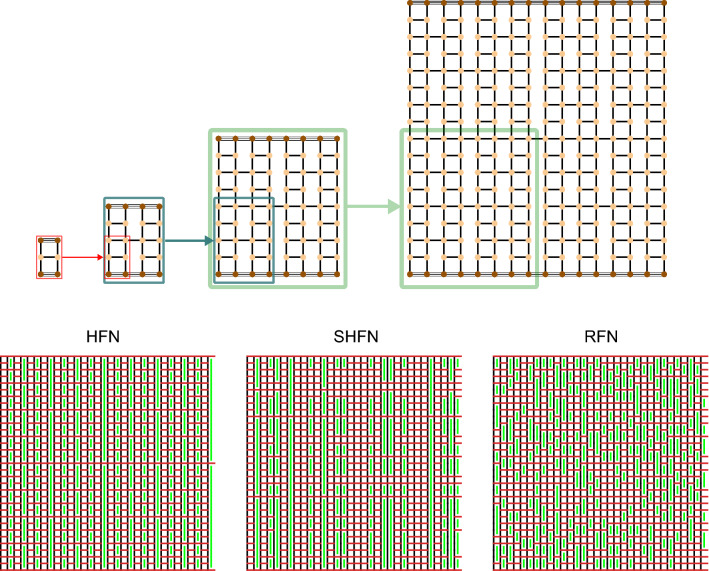
Network models. Top: Bottom-up construction of a HFN. A module of level zero is a load carrying vertical edge. A module of level 1 (generator) consists of 4 level-0 modules plus a load perpendicular cross link which spans the module. Higher level modules are constructed recursively by replacing in a module of level *n*, each level $n-1$ sub-module by a level-*n* module. The resulting structure defines a module of level $n+1$, as illustrated in the figure up to $n=4$. Circles indicate network nodes. Dark brown circles are boundary nodes, where boundary conditions are applied. Edges are represented as black segments connecting pairs of nodes. Boundary edges (triple segments) are not breakable and are excluded from the statistical GEBC study. Bottom: Examples of HFN, SHFN and RFN of $L=32$. Green lines indicate load parallel gaps^11,13^.

A (deterministic) hierarchical fuse network (HFN) of size $L=2^n$, where *n* is the number of hierarchical levels, can be constructed in an iterative “bottom-up” manner as shown in Fig. 1, top^11^. The resulting structure is sub-divided into a hierarchy of modules separated by load-parallel gaps (HFN panel in Fig. Fig. 1, green lines). Defining the length of a gap as the number of vertically adjacent missing cross links, we find that HFN exhibit a power-law type gap length distribution. From the point of view of GEBC, it is evident that this feature leads to high GEBC values in the cross links in between the longest gaps.

A non-deterministic version of a hierarchical network is obtained by starting from the HFN structure and then randomly reshuffling first the columns and then the rows of the adjacency matrix. This process, which preserves the power-law nature of the gap statistics and thus the hierarchical structure of the network, leads to a stochastic hierarchical fuse network, denoted as SHFN (Fig. 1, bottom center).

#### Random network construction

A RFN of size $L = 2^n$ contains the same number of load carrying edges and cross links as the corresponding HFN, but now the cross links are distributed randomly over the available pairs of horizontally adjacent nodes (Fig. 1, bottom). Alternatively, one may start from a lattice and randomly remove the same fraction of cross links that are missing in the corresponding HFN. This leads to a non hierarchical, statistically homogeneous structure where load parallel gaps have an exponential size distribution.

### Simulation protocol

**Figure 2:**
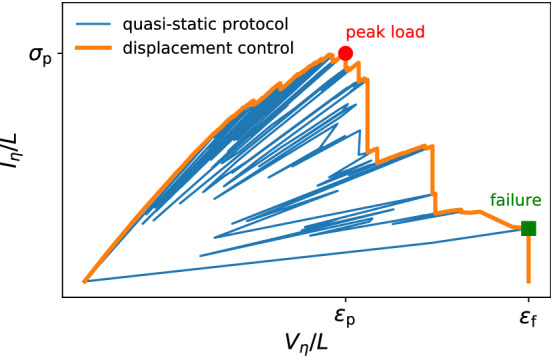
Example of $I-V$ characteristic. Thin blue line: data from the quasi-static simulation protocol. Thick orange line: displacement-control envelope, representing the dependence of the global stress $\sigma$ on the global strain $\varepsilon$ in the case in which the voltage difference between the top and bottom bus bars is increased monotonically. The peak stress $\sigma _\mathrm {p}$ denotes the peak load that the system can carry, while the failure strain $\varepsilon _\mathrm {f}$ stands for the maximum strain that is encountered in displacement control, before the system breaks. The interval between peak load and failure identifies the post-peak regime.

We simulate material loading and failure using the RFM. In this model the applied external potential resembles the mechanical displacement while the local current provides a measure of stress. Failure of network links occurs once the corresponding current exceeds a threshold value^32,33^. Our motivation to use the RFN is that this model not only is endowed with an evident network structure but has also been extensively studied for representation of basic features of failure processes in disordered materials^34^, including materials with hierarchical microstructures^11,12^. In the simulations, we follow the standard *quasi-static* deformation protocol^34^. We use an index $\eta$ to count the failing edges in the sequence of failure, starting from $\eta = 1$. To construct the failure sequence, a unit voltage difference is applied between the top and bottom bus bars, and all nodal voltages and edge currents are evaluated. At step $\eta$ one identifies the edge $(ij)_{\eta }$ with the highest load-strength ratio, $f_{\eta } = \max _{ij}(I_{ij}/t_{ij})$ and sets the global voltage $V_\eta$ to $f_{\eta }$, the value at which this critical edge fails. The corresponding global load is evaluated as the average current per upper or lower boundary edge, $I_{\eta } = f_{\eta } \sum _{ij\in {{\mathscr {B}}}} I_{ij}$ where the sum runs over the set ${{\mathscr {B}}}$ of *L* edges that connect the network to the top or bottom bus bars. The values of $f_{\eta }$, $I_{\eta }$, and $(ij)_{\eta }$ are stored. After setting the conductance of the critical edge to zero and increasing $\eta \rightarrow \eta + 1$, the computation is repeated to identify the next critical edge, etc. The process is terminated once the network is disconnected between top and bottom, as indicated by a zero global conductance. The final value $\eta = N_{\mathrm{f}}$ gives the total number of failed edges in the sample. The set of $(V_\eta ,I_\eta )$ pairs constitutes the stress-strain curve of the system – or $I-V$ characteristic – encoding its mechanical response to the applied load. A typical stress-strain curve is shown in Fig. 2 (thin blue line).

This simulation protocol mimics an idealized deformation process, where every time voltage is set to the exact value that produces failure of the weakest link (i.e. the link of highest load-strength ratio $f_\eta$), which ensures that only one link breaks at a time. From the sequence of link failures and the corresponding values of global voltage and current, the behavior under different, more realistic loading scenarios can be derived. For instance, one may assume that the system is loaded by monotonically increasing the voltage difference between the top and bottom bus bars – in what is known as *displacement control*. In this case, the $I-V$ characteristic and the sequence of broken links can be obtained from the quasi-static data by taking its voltage envelope, a procedure which we exemplify graphically in Fig. 2 (thick orange line)^34^. The new sequence of $V_{\eta _i}$ is the set of monotonically increasing values of *V* that we seek for the the displacement-control protocol, while the set of edges that are broken before the next increase in $V_{\eta _i}$ constitutes an *avalanche*. At every step, $\varepsilon =V_{\eta _i}/L$ is the global strain, and $\sigma =I_{\eta _i}/L$ the global stress. Similar considerations allow one to extract information for a scenario where the voltage difference is adjusted such as to impose an increasing global current through the network (*load control*).

In the present simulations, we consider the displacement-controlled scenario. Under this boundary condition, the global strain $\varepsilon$ always increases, the peak stress of the failure sequence is $\sigma _{\mathrm{p}} = \max _{\eta } I_{\eta }/L$, and the failure strain is $\varepsilon _{\mathrm{f}} = \max _{\eta } V_{\eta }/L = \max _{\eta } f_{\eta }/L$ (see Figure 2). In a typical simulation, the system first reaches the peak stress $\sigma _{\mathrm{p}}$ (and the corresponding strain value $\varepsilon _{\mathrm{p}}$) and successively fails (when strain reaches $\varepsilon _{\mathrm{f}}$). The peak stress signals an important deformation stage notably in non hierarchical RFN structures, where it separates a regime of stable, statistically homogeneous damage accumulation from a failure regime which is dominated by damage localization leading to nucleation and growth of a critical crack^34^. In HFN structures, instead, a system that has reached and passed peak stress still exhibits damage accumulation and prevents the formation of critical cracks, a fact that may result in an extended post-peak regime^11,12^.

## Results

### Evolution of edge betweenness centrality statistics

**Figure 3:**
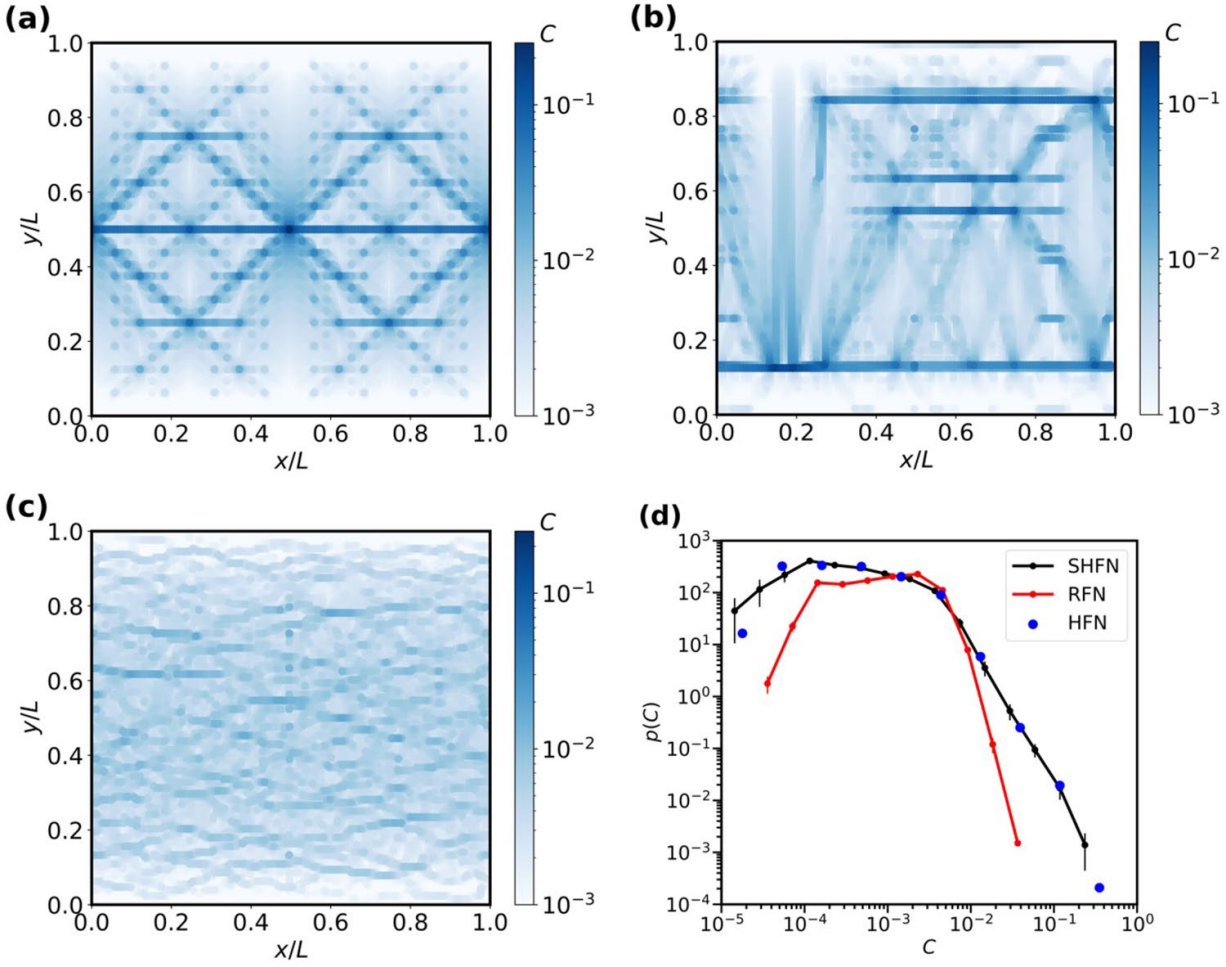
Distribution of geodesic edge betweenness centrality across the network. Network edges coloured based on GEBC values. (**a**) HFN model, (**b**) SHFN model and (**c**) RFN model, all of size $L= 128$, (**d**) probability distributions *p*(*C*) of edge betweenness centrality for the different network models. SHFN and RFN data are averaged over 200 network realizations. HFN and SHFN exhibit the same tail behavior.

In Fig. 3, the edge betweenness centrality patterns of different initially intact networks are plotted together with the associated GEBC statistics. Fig. 3a–c represent GEBC patterns where the edge midpoints are colored based on the edges *C* values.

Visual inspection of the patterns reveals distinctive differences between hierarchical and non hierarchical networks. In the hierarchical structures, GEBC is concentrated in the horizontal rows of cross links which connect multiple modules in the load-perpendicular direction. In these cross-links, GEBC can assume very high values. At the same time, it may be noted that these links are, in the initial undamaged state of the network, load free, i.e., the network structure systematically ensures that the most central links are not strongly loaded. In the random RFN reference structures, on the other hand, the distribution of GEBC values is much more homogeneous and no distinctive GEBC patterns can be identified.

The probability distribution *p*(*C*) of edge betweenness centrality is shown for each network type in Fig. 3d, where for the stochastic SHFN and RFN networks the statistics have been averaged over the initial conditions of 200 realizations. As expected from visual inspection of the GEBC patterns, the distribution of *C* values for RFN structures is much narrower than for their HFN and SHFN counterparts. Moreover, the distributions for HFN and SHFN exhibit a fat power-law tail towards high GEBC values where $p(C) \propto C^{-\delta }$ with $\delta \approx 2.6$, a behavior which reflects the power-law statistics of gap lengths in the hierarchical-modular structure.

**Figure 4:**
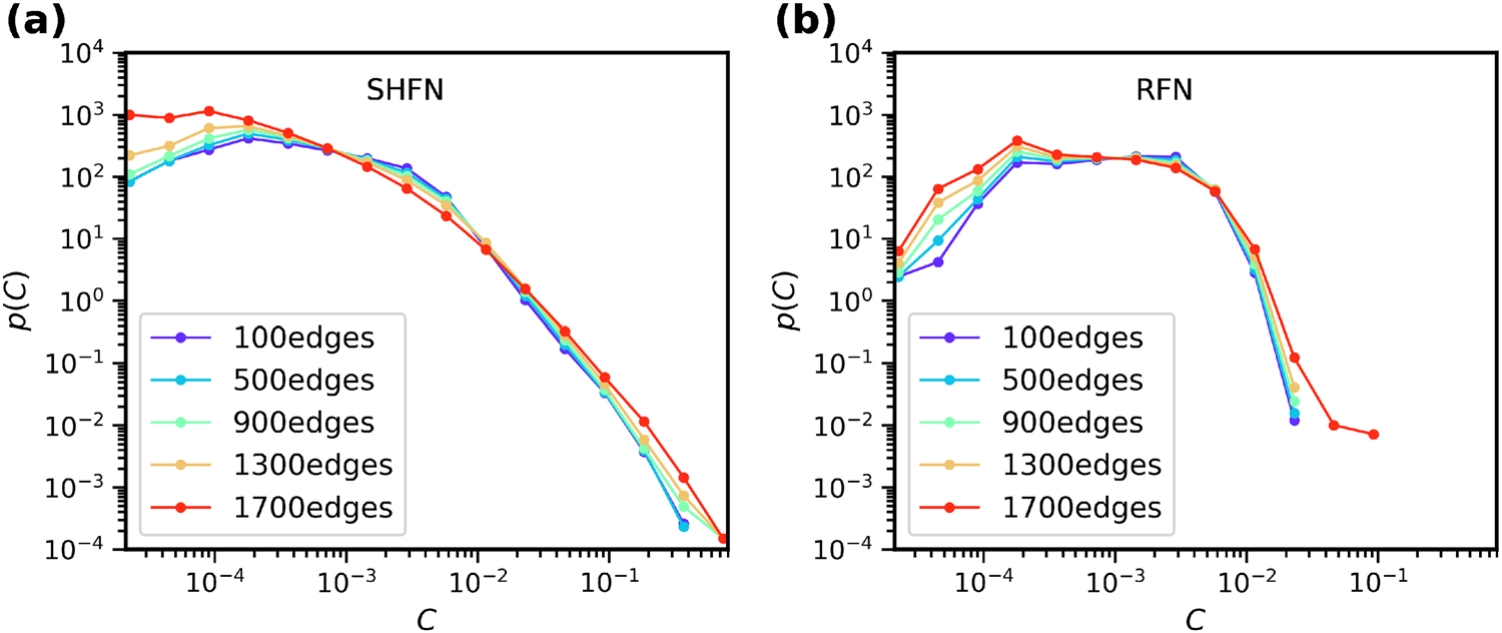
Evolution of GEBC statistics with accumulating damage. *p*(*C*) vs. *C* curves after 100, 500, 900, 1300 and 1700 failed edges, for hierarchical SHFN (**a**), and non-hierarchical RFN (**b**). In all simuations $L= 128, k=3$.

Under increasing load, accumulation of damage is accompanied by changes in the statistical distribution of GEBC. On the one hand, the probability of edge failure may depend on the edge’s *C* value, as suggested in the literature. On the other hand, the removal of an edge may change the GEBC values of other edges in the system. We have determined the evolution of the *p*(*C*) curve for different numbers of removed edges as shown in Fig. 4 for SHFN and RFN structures with $L=128$, for which we have determined *p*(*C*) curves after removal of 100, 500, 900, 1300 and 1700 edges. We note that the last number is close to global failure, which for the RFN structures occurs at about 1800 edge failures and for the SHFN at about 2100 failures.

For both SHFN and RFN structures, the evolution of GEBC statistics is characterized by a fattening of the distribution tails, at both low and high *C* values. The fattening of the high-*C* tail of the distribution is particularly evident in RFN where near failure, the *p*(*C*) probability density functions develop outliers that extend the spectrum of *C* values to much higher levels than in the initial state. The reason for this – at first glance surprising – behavior is that RFN fail by nucleation-and-propagation of a critical crack which separates the system into two parts^11^. Edges located near the crack tip thus acquire GEBC values which are much higher than any *C* values in the undamaged initial state and which increase with increasing crack length.

SHFN, on the other hand, fail by diffuse nucleation of damage without formation of a coherently propagating crack^11^. Here, the fattening of the high-*C* tail occurs in a gradual manner and without development of statistical outliers. Instead, we observe a slight decrease of the exponent $\delta$ as damage accumulates.

### Correlation between GEBC and failure propensity

Given that the GEBC statistics evolves in the run-up to global failure, when correlating GEBC and failure propensity we think it is mandatory to account for the stage of the damage process at which GEBC is determined, and the stage when failure occurs. We note in particular that failure in non-hierarchical systems (akin to our RFN model) is often interpreted as a critical phenomenon^34^, where scale-invariant behavior is encountered at peak load, e.g., in the form of avalanches that are power-law distributed in size. Hierarchical systems (and in particular our HFN and SHFN models), instead, exhibit *generic* critical-like behavior: while one can clearly identify a peak load at a given $\varepsilon _\mathrm {p}$, scale invariant avalanches are encountered in broad range of $\varepsilon <\varepsilon _\mathrm {p}$ for any system size^11^, a behavior which is was also highlighted in problems of hierarchical percolation^43,44^ and spreading^45^. Our aim is thus to analyze how the GEBC evolves with damage in this complex scenario.

For every simulation, we re-enumerate the failed edges based on their position $\beta _s = N_{\mathrm{f},s}-\eta _s$ in the failure sequence of sample *s*, counting backwards from the point of failure. Based on their $\beta _s$ values we divide the failed edges into classes ${{\mathscr {C}}}^\alpha = \cup _{s} \{z(\alpha -1) < \beta _s \le z\alpha \}$. Thus, each class consists of *zM* members where *M* is the number of simulated samples for a given set of parameters *L*, *k*. In the following we fix $z=25$, and in order to assess the role of system size we consider sizes $L=64,\,128,\,256$ and the corresponding numbers of realizations $M=400,\,200,\,50$. Thus class 1 ($\alpha = 1$) contains the last 25 edges to fail in all samples, class 2 the 25 edges in each sample to fail before class 1, etc.

**Figure 5:**
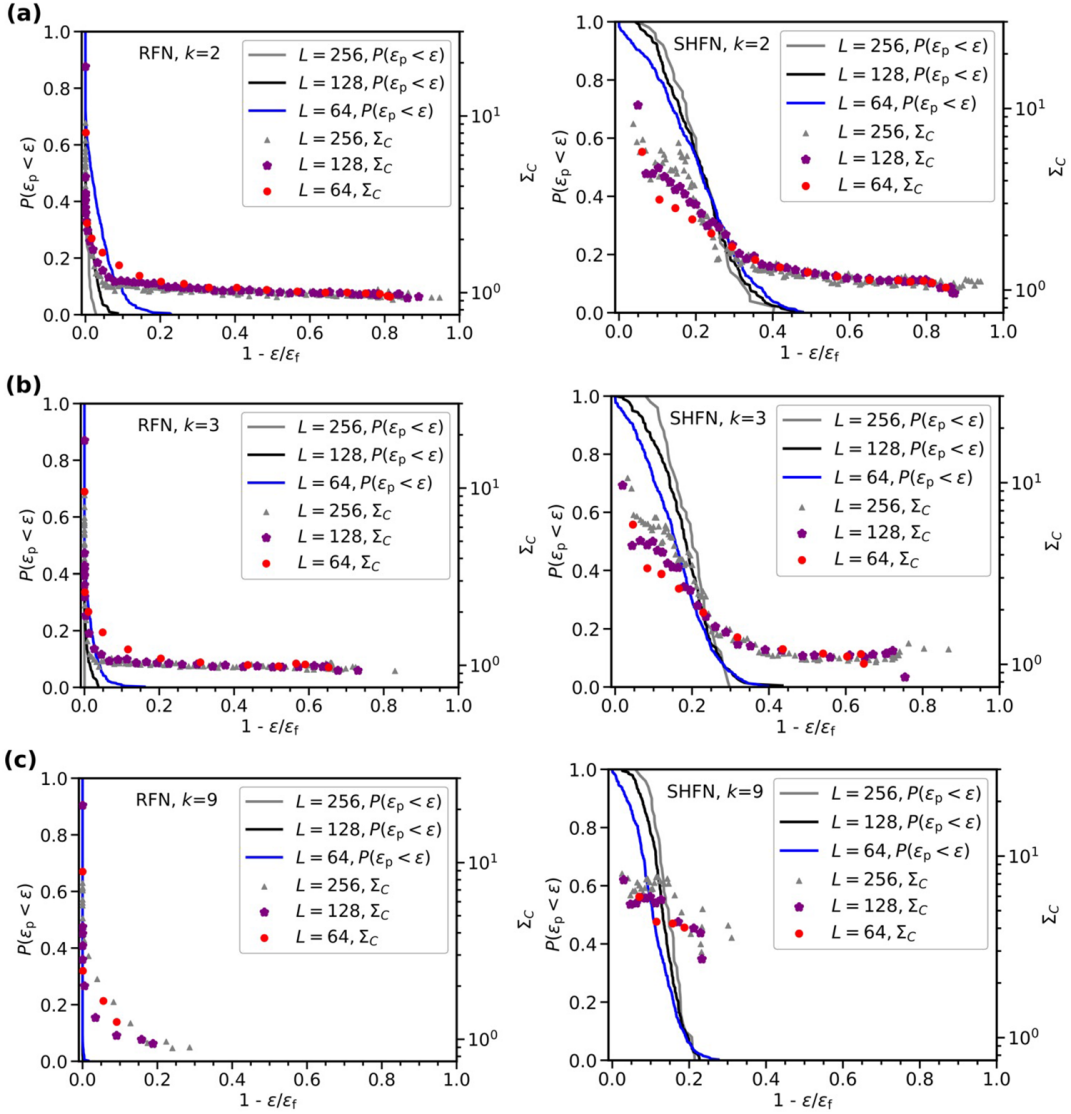
Correlation between GEBC and failure. Solid lines: Fraction of samples beyond the peak stress stage vs. reduced strain-to-failure, for networks of sizes $L=64,\,128,\,256$; left: RFN, right: SHFN; thresholds are Weibull distributed with shape factors $k=2$ (**a**), $k=3$ (**b**) and $k=9$ (**c**). Symbols: GEBC mean deviation vs. reduced strain-to-failure.

For each class $\alpha$ we compute the average ratio between edge failure strain and sample failure strain $\varepsilon /\varepsilon _\mathrm {f}$ and the fraction of samples that have passed their peak stress stage, $P(\varepsilon _\mathrm {p}<\varepsilon )$. Fig. 5 shows the dependence of $P(\varepsilon _\mathrm {p}<\varepsilon )$ on the strain-to-failure $1-\varepsilon /\varepsilon _\mathrm {f}$, where we recover the phase-transition-like scenario described above. Both RFN and SHFN display a transition behavior, where $P(\varepsilon _\mathrm {p}<\varepsilon )$ acts as an order parameter. This representation allows us to monitor the statistics of GEBC as follows. For the network configurations at the beginning of each class, we determine the statistics of GEBC values of all surviving edges in the simulated samples. The average of all the *C* values of sample *s* when the damage process is at the beginning of failure class $\alpha$ is denoted as ${\bar{C}}^{\alpha }_s$ and the values of the individual edges pertaining to class $\alpha$ as $C^{\alpha }_{\beta _s}$. Using these notations, we define class specific GEBC mean deviation $\Sigma _C^{\alpha }$ by

3 $$\begin{aligned} \Sigma _C^{\alpha } = \frac{1}{z M}\sum _s \sum _{\beta _s \in {{\mathscr {C}}}^\alpha } \left[ \frac{C^{\alpha }_{\beta _s}}{{\bar{C}}^{\alpha }_s}-1\right] . \end{aligned}$$

A zero value of $\Sigma _C^{\alpha }$ indicates that the edges failing in that class have, on average, the same GEBC as all edges in the sample, in other words, there is no correlation between GEBC and failure propensity. Positive values indicate that the failing edges have above-average GEBC, thus a positive correlation, whereas negative values demonstrate the opposite effect.

Figure 5 shows, for RFN and SHFN of different degrees of disorder, the evolution of the GEBC–failure correlations, by plotting for the different failure classes $\Sigma _C^{\alpha }$ values versus the respective mean strain-to-failure. A first comparison with the $P(\varepsilon _\mathrm {p}<\varepsilon )$ curves suggests that in all cases $\Sigma _C^{\alpha }$ increases rapidly as the system approaches the peak load stage (the increase in $P(\varepsilon _\mathrm {p}<\varepsilon )$: as damage progresses, GEBC exhibits higher correlation with failure propensity. Interestingly, while in RFN $\Sigma _C^{\alpha }$ clearly tends to an asymptotic behavior as sizes increase (especially in the lower *k* cases), it is mostly size-independent in the case of SHFN. The biggest deviations are encountered in the low disorder limit ($k=9$), where both RFN and SHFN reach failure after breaking very few edges, thus providing worse statistics for this type of study.

**Figure 6:**
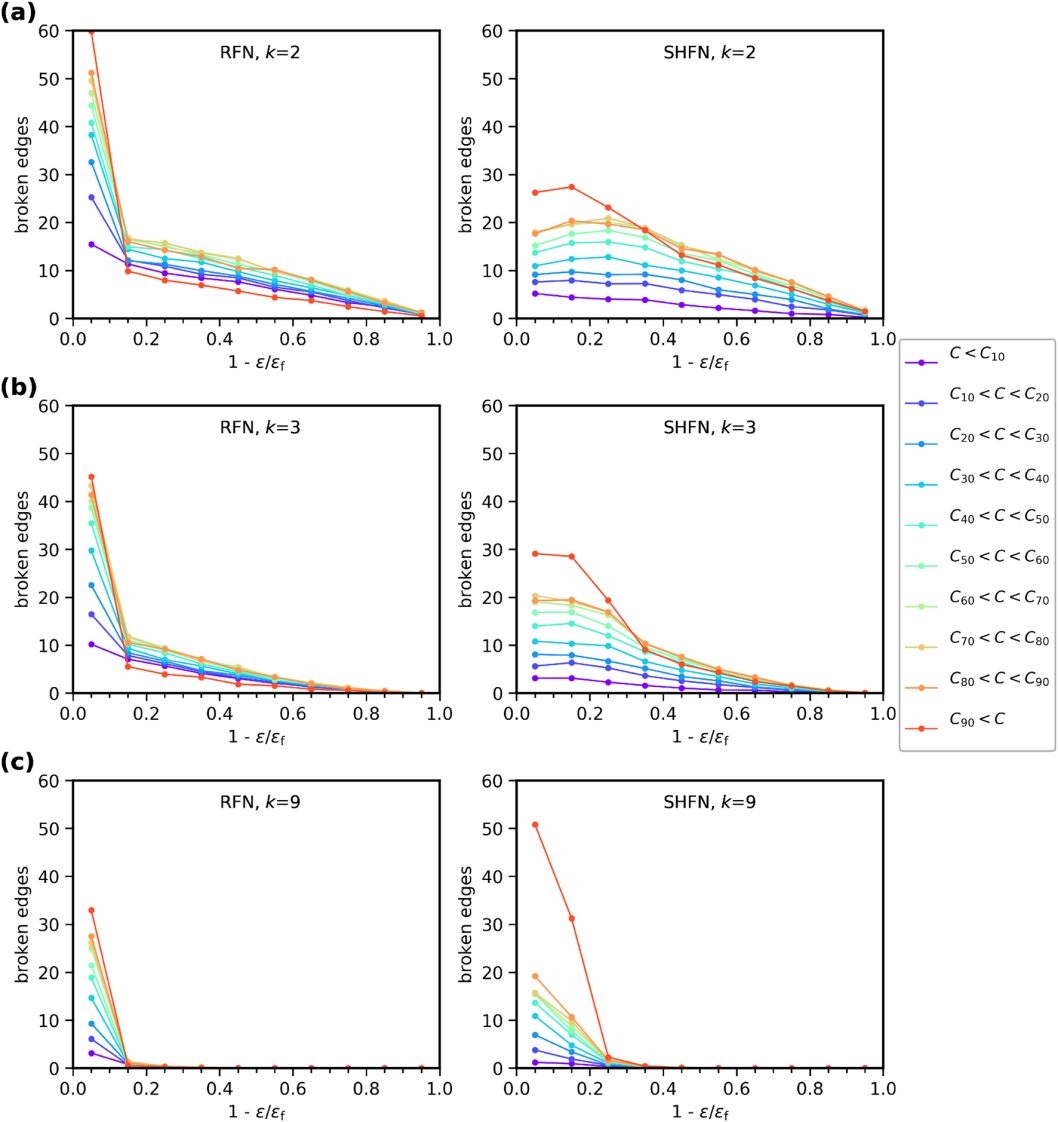
Edge failure predictions for RFN and SHFN, based on initial values of GEBC. Number of broken edges as a function of the reduced strain-to-failure variable, for systems of size $L=128$. Each curve represents the number of broken edges at every strain-to-failure stage, from the set of edges in the *i*th percentile of the distribution of initial GEBC, *p*(*C*). Thresholds are Weibull distributed with shape factors $k=2$ (**a**), $k=3$ (**b**) and $k=9$ (**c**).

**Figure 7:**
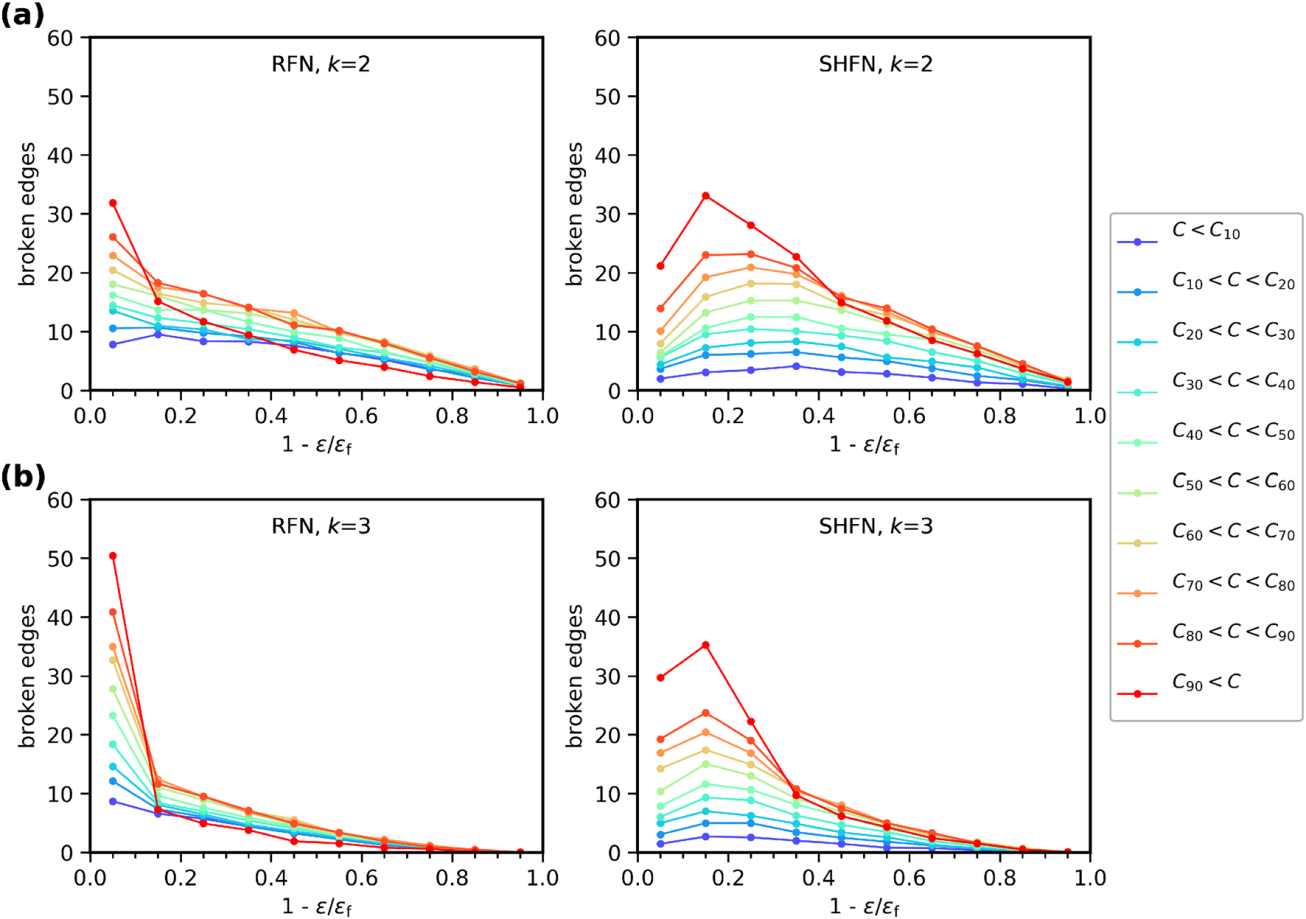
Edge failure predictions for RFN and SHFN, based on current values of GEBC. Number of broken edges as a function of the reduced strain-to-failure variable, for systems of size $L=128$. Each curve represents the number of broken edges at every strain-to-failure stage, from the set of edges in the *i*th percentile of the distribution of GEBC, *p*(*C*), recorded at the current strain-to-failure stage and reflecting the effects of accumulated damage. Thresholds are Weibull distributed with shape factors $k=2$ (**a**) and $k=3$ (**b**). Results for $k=9$ are not shown, as they do not differ significantly from those in Fig. 6(**c**).

To elucidate the correlations between GEBC and failure propensity in more detail, we study how the edges failing in each failure class distribute over the GEBC probability distribution. To this end, we divide the cumulative distribution of *C* into ten-percentiles $C_n$. Edges for which $C_{n-1} < C \le C_n$ then fall into the *n*th ten-percentile of the GEBC probability distribution. Similarly, we divide the strain-to-failure of a sample into ten-percentiles. We record for each strain ten-percentile the number of failed edges in each ten-percentile of the GEBC probability distribution. This is done in two different manners: Fig. 6 considers percentiles of the initial GEBC probability distribution prior to loading, it thus reflects the predictive value of the *initial* GEBC. Figure 7, by contrast, considers percentiles of the GEBC probability distribution at the beginning of the current strain interval, it thus accounts for changes in GEBC due to damage accumulation.

The curves in Fig. 6 show that the damage accumulation process is strongly influenced by disorder as reflected by the shape factor of the threshold distribution. Samples with high disorder (low *k*) show a gradual accumulation of damage which accelerates towards failure. In samples with low disorder, on the other hand, loading is mainly elastic and damage accumulation is concentrated close to the failure strain. Moreover, the behavior is more brittle in the sense that the total amount of damage accumulation is less (i.e., damage is more concentrated in a critical flaw). These effects are more pronounced in RFN than in hierarchical structures, which generally accumulate more damage before failure. These observations agree with the general picture of disorder-dependent damage and failure processes in hierarchical and non hierarchical structures as reported elsewhere^13,46^ where low disorder and absence of hierarchical structure promote a nucleation-and-growth scenario of brittle failure, whereas high disorder and hierarchical topology of the load carrying network promote diffuse accumulation of damage where failure occurs by damage percolation.

Turning to GEBC effects, we observe a general tendency that more edges fail in the upper percentiles of the GEBC probability distribution. As a exception to this general tendency, however, the highest GEBC ten-percentile accounts for the *smallest* amount of early damage accumulation in RFN structures, and also falls behind lower percentiles in SHFN structures. Only in the last stages of the failure process, typically beyond the peak stress stage, the highest GEBC ten-percentile dominates damage accumulation. This observation is valid irrespectively of whether one considers initial GEBC (Fig. 6) or current GEBC (Fig. 7).

## Discussion and conclusions

Our investigation confirms the finding of significant correlations between edge betweenness centrality in network structures and the propensity for edge failure under load. Such correlations can even be found in hierarchical structures that are architectured in such a manner that, in the absence of failed edges, the most central edges are load free and thus protected against failure.

At the same time, our investigation of the evolution of GEBC in the run-up to failure and of the associated correlations with failure propensity indicates that failure is very far from being controlled by network topology alone. There is a statistically significant global correlation between GEBC and failure propensity in the sense that failing edges tend to have above average GEBC, and this correlation actually increases in the run-up to global failure. However, this general correlation does not necessarily mean that the edges with highest GEBC are most likely to fail – as we have demonstrated, under certain conditions (non hierarchical structure, low disorder), edges from the highest 10-percentile of the GEBC distribution are actually *less* likely to fail than edges from the lower percentiles. This scenario changes in the immediate vicinity of global failure: due to the reduction of stress redistribution pathways, more central edges carry higher loads and become more exposed to failure. Thus, changes in the local load pattern and correlated evolution of the GEBC pattern lead to behavior that cannot be fully captured in terms of a single statistical signature.

When considering claims that GEBC may serve as a tool for *forecasting* failure locations, another critical remark must be made. A generic problem in predicting materials failure resides in the fact that the actually damaged or failed volume usually amounts to a very tiny fraction of the system volume. In our simulations, the number of failed edges amounts, at the point of global failure, to between $< 2\%$ of all edges (non hierarchical structures of low disorder) and $\approx 20\%$ of all edges (hierarchical structures of high disorder). This problem, which typically increases with sample size, implies that any test that is used as a forecasting tool must be very specific, otherwise the prediction will be swamped by false positives^47^. Taken as a single indicator, GEBC falls very short of this requirement. However, GEBC data might be one component of more complex prediction strategies based upon analysis of multidimensional data and their correlations using machine learning approaches^31,47^.

## Data Availability

The datasets generated and used during the current study are available from the corresponding author on reasonable request.
